# A Nonlinear Local Approximation Approach for Catchment Classification

**DOI:** 10.3390/e26030218

**Published:** 2024-02-29

**Authors:** Shakera K. Khan, Bellie Sivakumar

**Affiliations:** 1 Water Forecasting Team, Environmental Prediction Services Program, Bureau of Meteorology, Sydney, NSW 2010, Australia; shakera.khan81@yahoo.com.au; 2 Department of Civil Engineering, Indian Institute of Technology Bombay, Powai, Mumbai 400076, India

**Keywords:** classification, nonlinear dynamics and chaos, phase-space reconstruction, prediction, dimensionality, prediction accuracy measures

## Abstract

Catchment classification plays an important role in many applications associated with water resources and environment. In recent years, several studies have applied the concepts of nonlinear dynamics and chaos for catchment classification, mainly using dimensionality measures. The present study explores prediction as a measure for catchment classification, through application of a nonlinear local approximation prediction method. The method uses the concept of phase-space reconstruction of a time series to represent the underlying system dynamics and identifies nearest neighbors in the phase space for system evolution and prediction. The prediction accuracy measures, as well as the optimum values of the parameters involved in the method (e.g., phase space or embedding dimension, number of neighbors), are used for classification. For implementation, the method is applied to daily streamflow data from 218 catchments in Australia, and predictions are made for different embedding dimensions and number of neighbors. The prediction results suggest that phase-space reconstruction using streamflow alone can provide good predictions. The results also indicate that better predictions are achieved for lower embedding dimensions and smaller numbers of neighbors, suggesting possible low dimensionality of the streamflow dynamics. The classification results based on prediction accuracy are found to be useful for identification of regions/stations with higher predictability, which has important implications for interpolation or extrapolation of streamflow data.

## 1. Introduction

Catchment classification plays an important role in many hydrologic, environmental, and ecologic applications. Such applications include the following: (1) hydrologic regionalization for extrapolation of information [1,2,3,4,5,6]; (2) identification of model complexity [7,8,9]; (3) prediction and model parameterization in ungauged catchments [10,11,12,13,14,15,16,17,18]; (4) predictions under changed flow conditions [6,11,19,20,21,22]; (5) assessment of environmental flows [23,24,25,26,27]; and (6) eco-hydrologic classification [28,29].

A review of the literature suggests that catchments can be classified based on river morphology [30], river/flow regimes [1,25], hydrologic similarity indexes [3,5,6,14,16,18,31,32,33,34,35], hydroclimatic factors [11,36], ecohydrologic factors [28,33,37,38,39], and other factors. Many methods have been employed to use these bases for catchment classification, including regression-based methods [3,8,33,40], clustering [5,6,8,16,17,19,21,33,34,35,36], flow duration curve analysis [12,13,41], principal component analysis [3,6,18,29], and process-based modeling [11], among others. Applications of the concepts of community structure within the context of complex networks for catchment classification are also starting to emerge [42,43].

In recent years, there have been some attempts to use the concepts of nonlinear dynamics and chaos theory for catchment classification, following the encouraging outcomes reported by earlier studies on the applications of these concepts for various purposes in hydrology, including characterization, prediction, missing data estimation, and disaggregation of hydrologic time series; see [44] for a comprehensive account. Such studies on catchment classification have employed the phase-space reconstruction method [7], the correlation dimension method [8], and the false nearest neighbor (FNN) method [9]. For instance, [7] employed the phase-space reconstruction approach [45,46] to classify streamflow series from several rivers around the world based on their ‘complexity’. They used the ‘region of attraction of trajectories’ in the phase space to identify data as exhibiting ‘simple’ or ‘intermediate’ or ‘complex’ behavior and, correspondingly, classify the system as potentially low-dimensional, medium-dimensional, or high-dimensional. Sivakumar and Singh [8] employed the correlation dimension method [47], in addition to phase-space reconstruction, to estimate the dimensionality of monthly streamflow series from 117 stations in the western United States (US) and, hence, to classify the time series. The results indicated certain homogeneous regions in terms of the dimensionality of the flow series, with minor exceptions. Vignesh et al. [9] employed the false nearest neighbor method, a dimensionality-based approach, to examine spatial variability in a large network of 639 stations across the US and, hence, to offer some kind of classification based on the optimal embedding dimension. The FNN dimensions indicated a wide variation in the streamflow dynamics, but with a generally low level of complexity for most stations.

Encouraged by the outcomes of these studies, the present study uses a prediction-based nonlinear dynamic method, popularly known as the ‘Nonlinear Local Approximation Prediction Method’, for catchment classification. The method uses the concept of phase-space reconstruction of a time series to represent the underlying system dynamics and identifies nearest neighbors in the phase space for system evolution and prediction. The prediction accuracy measures, as well as the optimum values of the parameters involved in the method (e.g., phase space or embedding dimension, number of neighbors), are used for classification of catchments. To evaluate the suitability and effectiveness of this classification approach, daily streamflow data observed over a period of 24 years (1983–2006) from 218 catchments in Australia are considered. Embedding dimension (*m*) values from 1 to 10 are considered for phase-space reconstruction and subsequent predictions (one timestep ahead), and different numbers of nearest neighbors (i.e., based on minimum Euclidean distances in the phase space, which is an indication of ‘similarity’ in streamflow values), ranging from *k* = 1 to 500 are also considered. The accuracy of predictions is evaluated in terms of four statistical measures: normalized root mean square error (NRMSE), correlation coefficient (CC), coefficient of determination (R^2^), and Nash-Sutcliffe efficiency (NSE), along with other measures, such as the direct time series and scatter plots.

The organization of the rest of this paper is as follows. Section 2 presents a brief description of the nonlinear local approximation prediction method. Section 3 describes the study area and data used in this study. Section 4 presents the nonlinear prediction analysis and results of streamflow time series, and their classification using the prediction results. Section 4 draws some conclusions and also offers potential directions for further research.

## 2. Materials and Methods

### 2.1. Methodology

In this study, a nonlinear local approximation method is applied to time series (streamflow) from various locations (stations), and the prediction accuracy measures are then used for classification of catchments. The nonlinear local approximation method involves phase-space reconstruction of time series and prediction of system evolution in the reconstructed phase space. The method is described next.

Phase space is essentially a graph or a co-ordinate diagram, whose coordinates represent the variables necessary to describe the state of a system at any given time [45]. The trajectories of the phase-space diagram describe the evolution of the system from some initial state (which is assumed to be known) and, hence, they represent the history of the system. Given a single-variable (or multi-variable) series *X_i_*, where *i* = 1, 2, …, *N*, a multi-dimensional phase space can be reconstructed according to the method of delays [46] as follows:

***Y**_j_* = (*X_j_*, *X_j+τ_, X_j+2τ_*, …, *X_j+(m–1)τ_*_/∆*t*_) (1)

where *j* = 1, 2, …, *N* − 1(*m* − 1)τ/∆*t*; *m* is the embedding dimension of the vector ***Y**_j_* (normally considered to represent the minimum number of variables required to describe the system), and *τ* is the delay time usually chosen as a suitable integer multiple of the sampling time ∆*t*. There are many different methods and ways to estimate the optimal embedding dimension. An accurate representation of the phase space also requires selection of an appropriate delay time, which can be determined using, for example, the autocorrelation function method [48,49] and the average mutual information method [50].

The nonlinear prediction (NLP) method was originally developed by Farmer and Sidorowich [51] and has been used in many studies for prediction of hydrologic time series and identification of chaos [52,53,54,55,56]. Once the phase space is correctly reconstructed in a dimension *m* for a (single or multi-variable) series, the non-linear dynamics of a system can be represented in terms of an *m*-dimensional mapping function, *ƒ_T_*, written as:

***Y****_j_*_+*T*_ = *f_T_* (***Y****_j_*) (2)

where ***Y****_j_* and ***Y****_j_*_+*T*_ are vectors describing the state of the system at times *j* (current state) and *j* + *T* (future state), respectively. The lead time *T* can be chosen (normally an integer multiple of the timestep of the time series) in such a way as to predict any future state. For chaotic systems, however, reliable predictions are, in general, possible only for small lead times, and the prediction accuracy dramatically decreases with further increases in lead time.

An appropriate expression for *f_T_* (e.g., *F_T_*) can be found to predict the future using either global or local approximation techniques. The global approach approximates the map by considering the entire phase space of the attractor in quest for a valid form for all points (e.g., neural networks and radial basis functions). On the other hand, in the local approximation approach, only nearby states are used to make predictions, and the dynamics are modeled locally and individually in the embedding space [51].

In the local approximation approach, the *f_T_* domain is subdivided into many local neighborhoods, and an approximation for *F_T_* is made for each neighborhood, which is valid only in that neighborhood. In this way, the system dynamics are characterized locally piecewise in the phase space, which reduces the complexity in modeling *f_T_* without affecting the accuracy of prediction. To predict the value of the variable *X* at a future time *X_j_*_+*T*_ using past observations and an *m*-dimensional vector ***Y****_j_* at the current state, *k* nearest neighbors of ***Y****_j_* are found based on the minimum values of ║***Y****_j_* − ***Y****_j_*′║, where *j*′ < *j*. In this study, the basis for selection of the nearest neighbor(s) *k* is the Euclidean distances between the reconstruction vectors. More specifically, if the Euclidean distance between two reconstructed vectors (among all the reconstructed vectors of interest, i.e., with respect to the last vector) is the minimum and especially if it is very small, then this means that these two vectors are very similar, which can then help determine the evolution.

When the prediction is made using a single neighbor, the prediction of *X_j_*_+*T*_ would be *X_j_*_′+*T*_. For *k* number of neighbors, the prediction of *X_j_*_+*T*_ could be made by taking an average of the *k* values of *X_j_*_′+*T*_ (i.e., equal to $\frac{1}{k}\;\sum_{i=1}^{k}X_{j’+T}$). An optimum value of *k* is determined by a trial-and-error procedure [54,55]. The accuracy of prediction can be evaluated in terms of several statistical measures. In the present study, the normalized root mean square error (NRMSE), correlation coefficient (CC), coefficient of determination (R^2^), and Nash-Sutcliffe efficiency (NSE) are used. In addition to these statistical measures, direct time series plots and scatter diagrams are also used to evaluate the accuracy of prediction and to identify the optimum embedding dimension and number of neighbors. A brief description of the above four statistical measures is presented in Appendix A.

### 2.2. Study Area and Data

In the present study, the streamflow time series from a large number of streamflow monitoring stations in Australia are used for the purpose of classification of the catchments. Australia is the driest among all the continents, having a low annual rainfall (with a mean of 451 mm/year), high mean annual temperature (21.5 °C), high evaporation rate, and low annual runoff (with a mean annual runoff coefficient of 12%) [25,57]. The continent experiences a wide range of climatic conditions, with a large part of central and western Australia being characterized by an arid and semi-arid climate, the northern region by a tropical climate, and the south-eastern and south-western regions showing a temperate climate [58,59,60]. Australian catchments are generally characterized by their low topography (mean elevation is 330 m above sea level with a maximum elevation of 2745 m above sea level), typically ‘peakier’ flows, low base flows, smaller runoff coefficients, elongated dry periods, and large temporal variations in runoff [25,61,62].

**Figure 1:**
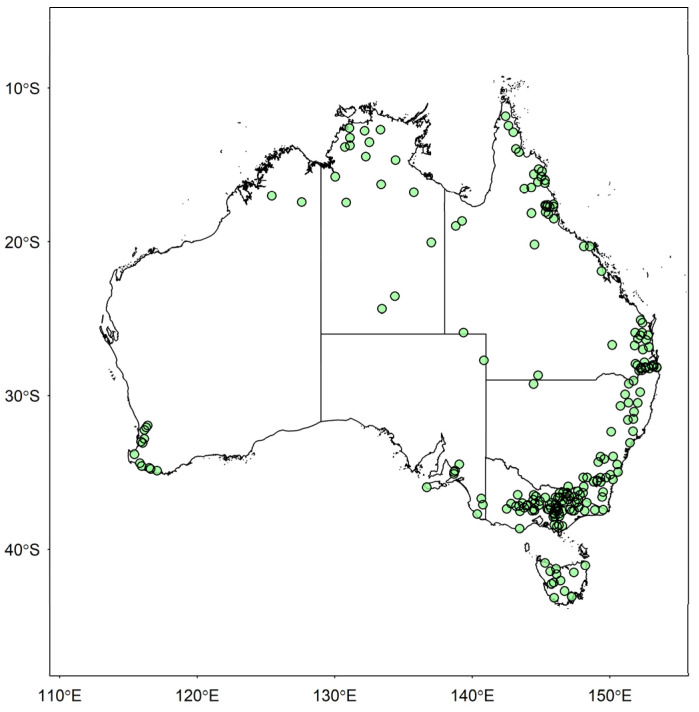
Locations of the 218 Australian streamflow stations used in this study.

In this study, streamflow time series from 218 stations in Australia are analyzed for catchment classification. These stations are defined by the Bureau of Meteorology, Australia as “hydrologic reference stations” (HRS). The characteristics of the HRS include the following: (i) unregulated or minimally affected by construction of dams, weirs, and other irrigation infrastructures, land use changes, bushfires, etc.; (ii) having a record length of at least 30 years and at least 15 years of continuous data in each climatic region; (iii) spatially and temporally distributed across all hydro-climatic regions in Australia; and (iv) having less than 5% missing data, and high-quality stage and rating curves for all aspects of flow regime. The stations considered in this study represent the major climatic zones, jurisdictions, and most of the drainage divisions in Australia. Figure 1 shows the geographic distribution of these stations. Table 1 summarizes the state-wide distribution of these 218 stations, along with their climatic zones, catchment areas, record lengths, elevations, and stream lengths.

In this study, streamflow data observed over a period of 24 years (1983–2006) from each of the 218 stations are analyzed. Daily streamflow data are used, since only finer timescales can appropriately represent the actual response of the climate-driven hydrology of Australia [62]. All data available for these stations are compiled and quality assured by the Bureau of Meteorology, Australia, and, therefore, are consistent nationally and over time [63]. Table 2 presents some important characteristics of the daily streamflow data from the 218 stations along with their corresponding station IDs.

**Figure 2:**
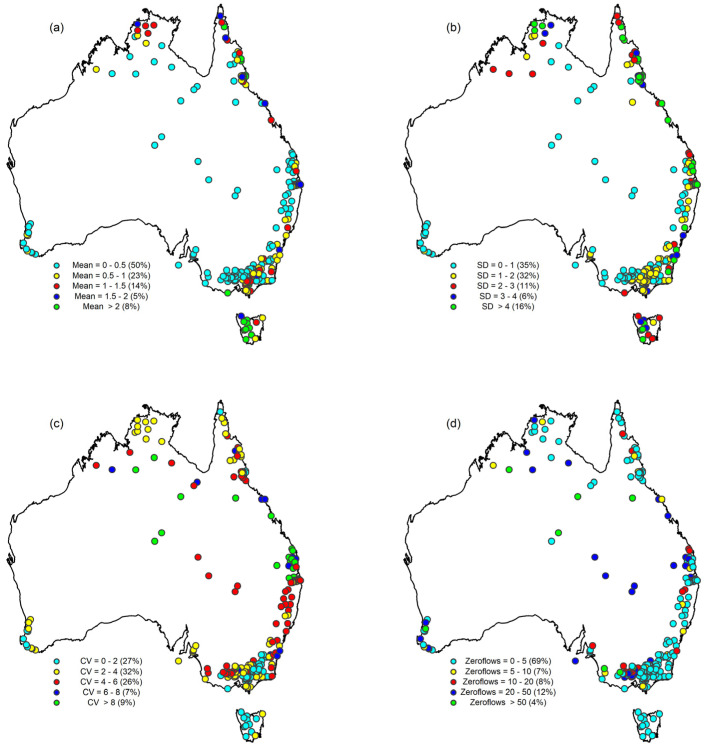
Daily streamflow statistics of the 218 stations used in this study: (**a**) Mean (MLD); (**b**) Standard deviation (MLD); (**c**) Coefficient of variation; and (**d**) Percentage of zero flow days (values inside parentheses represent the percentage of stations, rounded off to the nearest integer).

Figure 2 shows the spatial variations in the mean, standard deviation, and coefficient of variation of flow, as well as the number of zero flow days, for the 218 stations. The plots reveal that stations with relatively low daily mean flows (less than 0.5 MLD) are located mainly, and in varying degree, along the north-eastern, south-eastern, and south-western coasts, Northern Territory, and inland areas, while stations located across the north-eastern coast and Tasmania show relatively higher daily mean flows (more than 1.5 MLD). The coefficient of variation of daily flows is generally lower for stations located in the south-eastern coast, south-western coast, and Tasmania, while it is higher for stations in the north-eastern coast, Northern Territory, and inland areas. Stations located in the Northern Territory, north-eastern coast, south-eastern coast, and Tasmania have the lowest percentage of zero flow days (less than 5%), while the highest percentage of zero flow days is observed mostly in the inland areas, south coast, and south-western coast.

## 3. Results and Discussions

In this study, the nonlinear local approximation method is applied to predict the streamflow dynamics of 218 catchments in Australia and, subsequently, to classify the catchments based on the prediction results. Daily streamflow data over a period of 24 years from 1983 to 2006 are considered. The first 19 years of data (about 80%) are used for phase-space reconstruction, and predictions are made for the remaining 5 years of data (about 20%). Embedding dimension (*m*) values from 1 to 10 are considered for phase-space reconstruction and subsequent predictions, and predictions are made one timestep ahead (i.e., 1 day). A delay time of *τ* = 1 is used for phase-space reconstruction, since this is the minimum possible value and likely to provide the best reconstruction (i.e., highest correlation between the successive elements in the reconstructed vectors) for prediction purposes. Different numbers of nearest neighbors (*k*) are also considered. Taking into consideration the computational time and resources required, and also the differences possible in predictions (i.e., extent of improvement with increase in the number of neighbors), a total of 15 specific *k* values within the range of 1 to 500 are considered: (*k* = 1, 2, 3, 4, 5, 10, 20, 50, 75, 100, 150, 200, 300, 400, and 500). For the purposes of illustration and discussion here, detailed analyses and results are presented for only one station (station ID #401012, Murray River at Biggara, NSW). However, similar results were produced also for the remaining 217 stations. The prediction results for all 218 stations are then used for the classification of the catchments.

### 3.1. Prediction of Streamflow Series from Station ID #401012

**Figure 3:**
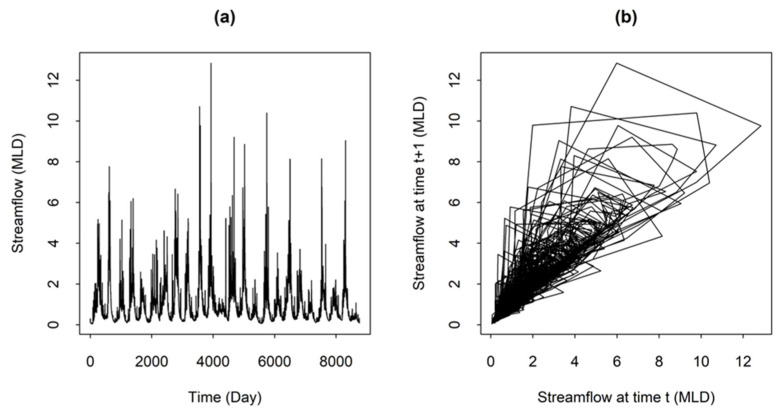
Daily streamflow from Station #401012: (**a**) Time series; and (**b**) Phase space diagram.

Figure 3a and b show the daily streamflow time series and phase-space diagram, respectively, for Station #401012. The phase-space diagram is reconstructed according to Equation (1) in two dimensions (*m* = 2) with a delay time *τ* = 1, so that the projection of the attractor on the plane is {*X_i_*, *X_i_*_+1_}. In Figure 3b, the connected points of trajectories show the evolution of the system and are somewhat scattered all over the phase space. The diagram shows that the shape of the attractor for this streamflow time series is reasonably well structured within a fairly narrow region of the phase space, indicating that a reasonably clear attractor is still present with intermediate level of system complexity, and the system is potentially medium-dimensional. This seems to suggest that the local approximation-based method can be useful to obtain reliable predictions.

**Figure 4:**
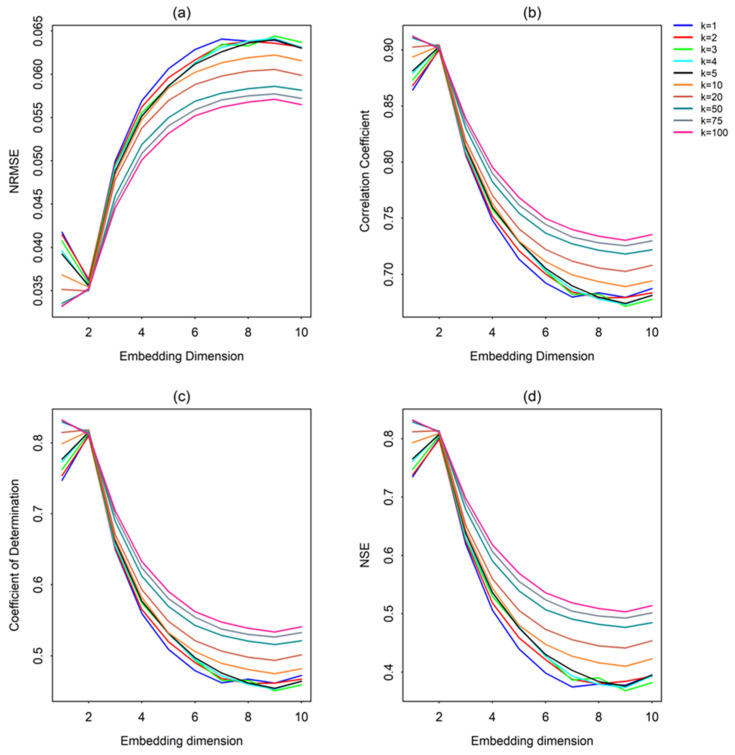
Prediction accuracy measures against embedding dimension for streamflow series from Station #401012: (**a**) NRMSE; (**b**) Correlation coefficient; (**c**) Coefficient of determination; and (**d**) Nash-Sutcliffe efficiency.

As mentioned earlier, the accuracy of the nonlinear prediction is evaluated in terms of four statistical measures: normalized root mean square error (NRMSE), correlation coefficient (CC), coefficient of determination (R^2^), and Nash-Sutcliffe efficiency (NSE). Figure 4a–d present these four prediction accuracy measures against the embedding dimension for the streamflow series from Station ID #401012 for 10 of the 15 different *k* values considered in this study (Results for *k* > 100 are not presented, as there is almost no improvement).

As seen from Figure 4, the prediction accuracies are higher for lower embedding dimension values (up to *m* = 2), gradually deteriorate when the embedding dimension is increased up to a certain point (up to *m* = 6 to 8, as appropriate for different *k*), and finally reach some kind of saturation (or only slightly improve) beyond that point. These generally low m values yielding better predictions seem to suggest the possible presence of chaos in the streamflow time series [64,65]. The deterioration of the prediction results at embedding dimensions higher than 2 is more likely to be an indication of the influence of the presence of noise in the data, as noise propagates more at higher dimensions (i.e., greater than the optimal dimension) and, hence, leads to declining prediction accuracy. Similar results (i.e., low m values yielding better predictions) have also been reported by many past studies on streamflow and other hydrologic data from other parts of the world [52,55,65].

It may appear, in some cases shown in Figure 4, that a higher embedding dimension or a higher number of neighbors yields better prediction accuracy estimates than for lower *m* and *k*. However, a higher correlation coefficient or a lower NRMSE value for a given *m* or *k* value does not always guarantee that the best prediction is achieved, and that a particular *m* and *k* value may not necessarily be the optimum m (*m_opt_*) or optimum *k* (*k_opt_*), due to a possible averaging effect for higher embedding dimensions or higher number of neighbors in the neighbor search technique. For example, as seen in Figure 4, when using only one neighbor for the prediction (i.e., when *k* = 1), improved prediction accuracy for higher embedding dimensions may imply that better prediction is achieved by considering the history of several dominant variables (e.g., rainfall, temperature, etc.) in the reconstruction, which significantly affects the history of streamflow. This statement is based on the following hypothesis: a non-linear system is characterized by self-interaction, and a single variable phase-space reconstruction can deliver all the information necessary to describe an entire multi-variable system by incorporating additional historic time steps (i.e., increased embedding dimensions by considering the influence of other dominant variables).

**Figure 5:**
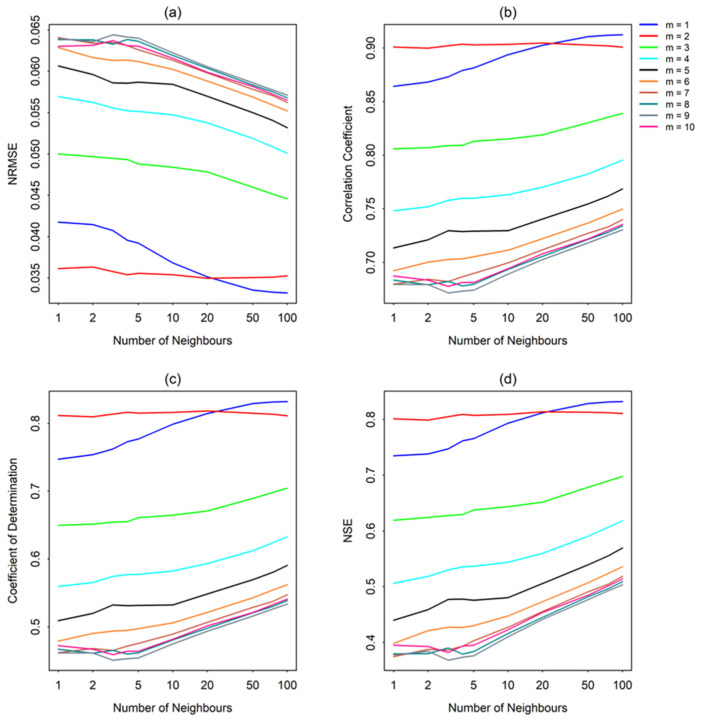
Prediction accuracy measures against number of neighbors for streamflow series from Station #401012: (**a**) NRMSE; (**b**) Correlation coefficient; (**c**) Coefficient of determination; and (**d**) Nash-Sutcliffe efficiency.

Figure 5a–d show the four prediction accuracy measures against the number of neighbors for different *m* values (i.e., m from 1 to 10). The plots show that the prediction accuracy, in general, slightly improves with an increase in the neighborhood size up to *k* = 5, and then there is a relatively rapid improvement for higher *k*, which, nevertheless, is still small enough considering the total number of neighbors. These results (i.e., a relatively small providing the best predictions) also indicate chaotic behavior in the time series [66,67]. This behavior for the streamflow time series from Station #401012 is also consistent with the results reported by past studies for streamflow and other hydrologic data from other regions of the globe [65,68].

While investigating the prediction accuracy measures against different embedding dimensions and numbers of neighbors and subsequent identification of *m_opt_* or *k_opt_* may turn out to be challenging at times, prediction results can be used to identify, as is consistent with the embedding theory and predictions, the *m_opt_* and *k_opt_* values to adequately represent the underlying dynamics of the streamflow series. The *m_opt_* and *k_opt_* values are determined by carefully examining the prediction results for different combinations of *m* and *k*. For example, the *m_opt_* or *k_opt_* value is the one that yields the lowest normalized root mean square error (NRMSE) as well as the highest correlation coefficient, coefficient of determination, and Nash-Sutcliffe efficiency. As an additional check and confirmation, time series and scatter plots for all possible combinations of *m* and *k* are also examined to assist in identifying *m_opt_* and *k_opt_*.

**Figure 6:**
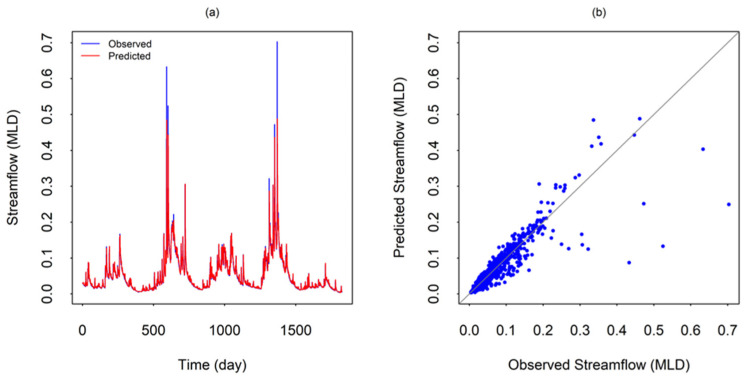
Comparison of predicted and observed streamflow values for Station #401012: (**a**) Time series; and (**b**) Scatter plot. The predicted values correspond to those obtained using embedding dimension (*m*) = 1 and number of neighbors (*k*) = 50.

Based on the four prediction accuracy measures, as well as the comparisons using time series and scatter plots, for the daily streamflow series from Station #401012, the *m_opt_* value is identified as 1 and the *k_opt_* value is identified as 50. Figure 6 shows the comparison between the predicted values obtained using the above *m_opt_* and *k_opt_* values and the observed values through time series plots (Figure 6a) and scatter plots (Figure 6b). As seen, the local approximation prediction method well captures the major trends and fluctuations of the time series, although there is certain underprediction (significant, in some cases) of extreme events. The scatter plot also suggests that phase-space reconstruction using streamflow alone provides reasonably good predictions for most streamflows, but significant underpredictions are also observed in a few cases. Inclusion of other key governing variables (e.g., rainfall, evapotranspiration) in the phase-space reconstruction, in a purely multi-variable phase-space reconstruction sense, can provide more accurate predictions. Such an exercise, however, is beyond the scope of the present study.

### 3.2. Classification of 218 Catchments Using Nonlinear Prediction Results

The procedure explained in the previous section is applied to each of the remaining 217 stations to obtain the optimum embedding dimensions and the best prediction accuracy measures, which are then used for classification of all 218 stations. The number of classes and the ranges of prediction accuracy measures are chosen somewhat arbitrarily. Based on the minimum and maximum values of each of the prediction accuracy measures, the stations are divided into the same number of classes (for all measures) for consistency and comparison.

**Figure 7:**
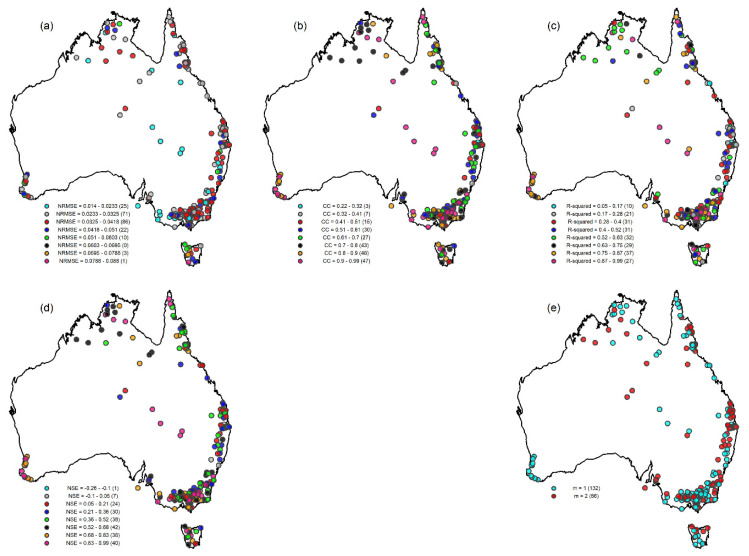
Classification of 218 stations using nonlinear prediction results for streamflow series: (**a**) Classification based on NRMSE; (**b**) Classification based on CC; (**c**) Classification based on R^2^; (**d**) Classification based on NSE; and (**e**) Classification based on optimal embedding dimension (*m_opt_*).

Figure 7 presents the classification of the 218 stations based on the four prediction accuracy measures (Figure 7a–d) and the optimum embedding dimensions (Figure 7e). Considering the NRMSE-based classification (Figure 7a), for example, the results indicate that stations with the lowest NRMSE values are mainly located across the inland areas near the border between Queensland and South Australia, southern coast, south-east coast, and a few in the eastern coast, and the far north of Western Australia. Stations located in the south-west coast, Northern Territory, and the eastern part of Australia show low to medium ranges of NRMSE values. Stations in Tasmania are mostly less predictable in terms of their prediction measures. When the classification is done based on correlation coefficient (Figure 7b), results show that stations in the Northern Territory, south-west coast, south-east coast, north-east coast, and Tasmania show relatively higher ranges of CC, while low to medium ranges of CC are observed for eastern-coast stations. Similar results are obtained for classification based on NSE (Figure 7d). For R^2^-based classification (Figure 7c), stations with the lowest NRMSE values are mainly located across the eastern coast, whereas the south-west coast stations show higher R^2^ values, and most of the stations across the Northern Territory, south-east coast, and Tasmania show medium to high ranges of R^2^ values.

As for the classification based on embedding dimension (Figure 7e), all stations in the south-west coast region have an optimal dimension of 1 (i.e., dominated by only one influencing variable). In the Northern Territory, most stations have *m_opt_* = 1, with a few stations having *m_opt_* = 2. Stations located in the eastern part of Tasmania have a mixture of *m_opt_* = 1 and *m_opt_* = 2, with most of the stations in the south-east coasts having *m_opt_* = 1. The results also show that more than half of the stations (about 60%) have *m_opt_* = 1, and only a few stations have *m_opt_* = 2.

As seen from Figure 7, use of different prediction evaluation measures and optimal embedding dimension as bases for classification can yield different classification of the catchments. Therefore, among these, the best one for classification is hard to identify. However, if the predictability is interpreted in terms of the complexity of the system, which, in turn, depends on the number of variables dominantly governing the system dynamics, then classification based on dimensionality (optimal embedding dimension) may be particularly useful. It should also be noted that, in this study, the optimal embedding dimension is identified as the dimension that yields best prediction. There exist several other approaches to determine the optimal embedding dimension, including the correlation dimension method and false nearest neighbor algorithm; see [8,9] for their applications for catchment classification.

### 3.3. Significance of Classification Results

The results obtained from the present analysis for the 218 streamflow series from Australia suggest the suitability of the nonlinear local approximation prediction method for reliable streamflow predictions and, subsequently, for catchment classification. The results also offer useful information from streamflow series as to which station or even region is more predictable (and perhaps less complex) and which are less predictable (and perhaps more complex), and thus facilitate better classification of the streamflow series/region. This kind of information is important for selection of suitable stations/regions for interpolating information (e.g., data) within regions, or extrapolating to nearby regions for streamflow predictions, including in the context of predictions in ungauged basins.

It can be observed, from the classification results based on embedding dimensions, that stations located in the same geographic or climatic region may exhibit somewhat different properties (e.g., variability), requiring additional information or variables to reveal the nonlinearity in their dynamics. Therefore, “regionalization” of the stations based on their dimensionality (and other properties) is somewhat difficult. This observation is consistent with the findings of many previous studies, especially those that have applied nonlinear dynamic methods and complex networks [8,9,69,70]. However, since the optimum embedding dimension for each of the classes identified above represents the number of dominant variables influencing the streamflow dynamics, such classification based on dimensionality has important implications in the context of streamflow process or model complexity, and for catchment systems at large. Therefore, the above classification based on dimensionality (which, in turn, is obtained from nonlinear local approximation prediction) can suitably identify the type of data and model requirements for each class of catchments, instead of generalization of a particular type of model for all catchments irrespective of their needs.

The classification framework presented in this study is also aimed at helping modelers to identify suitable catchments to apply their models to and helping users to identify suitable models for their catchments. The effectiveness of the proposed classification framework can be verified by simulating the hydrologic outputs for different complexity levels of a selected hydrologic model and linking them to the properties of the catchments of interest. For example, the Australian Water Balance model (AWBM) [71] can be used to simulate hydrologic outputs by varying its level of complexity and comparing them against the complexities of the observed data. This will help to identify the appropriate model complexities for the catchments and match them against the catchment groups or subgroups obtained using the proposed method of classification.

## 4. Conclusions

The present study examined the usefulness of the nonlinear local approximation prediction method for catchment classification. The method was implemented on 24 years of daily streamflow time series from each of 218 stations in Australia. The accuracy of prediction was evaluated using four statistical measures and the results were used for classification. The optimum embedding dimension, which represents the number of variables dominantly governing the system dynamics, was identified through these prediction accuracy measures as well as the direct time series plots and scatter plots. The optimum embedding dimensions obtained for the 218 stations were also used for their classification.

The classification results presented here indicate variations in the degree of predictability and dimensionality among the stations, and indeed even for the neighboring catchments from the same geographic region. This indicates that geographic proximity is not always the best basis for classification; the intrinsic catchment properties and their nonlinear dynamic properties, rather than their geographic proximity, play a vital role for classification of catchments. Many past studies [8,9,69,70] have also reported similar findings, and this observation has important implications for interpolation or extrapolation of information from gauged to ungauged catchments. The study also identified optimum embedding dimensions (i.e., dimensionality) for different classes of catchments, which are useful for selecting the suitable model type and data requirements for hydrologic studies of these catchments.

The classification scheme presented in this study is important not only for understanding the streamflow variability among catchments, but also for identifying the predictability of catchments for extrapolation/interpolation purposes, with the assumption that catchments which are more predictable in their characteristics will offer better prediction accuracy when used as donor catchments. It is important to note, however, that this study has focused mainly on identifying the dimensionality of catchments in order to assess the degree of complexity of model and, therefore, no attempts were made towards identifying the chaotic or stochastic nature of the time series for selecting the type of model. Nevertheless, the dimension estimates provide important information about, and better understanding of, the streamflow dynamics of Australian catchments.

Although the present study has established the suitability of the nonlinear local approximation-based prediction method for catchment classification, there remain some important issues that need to be addressed for even better and more reliable outcomes. These include the following: (1) extension of the single-variable phase-space reconstruction to a multi-variable form by including other additional and relevant variables, such as rainfall, potential evapotranspiration, soil moisture, and temperature; (2) investigation of the effects of other temporal scales (e.g., monthly timescale) on nonlinear prediction results; (3) exploration of the relationship between the prediction results and catchment physical properties, which is important for a better understanding of the catchment dynamics; (4) study of the effects of delay time in the phase space reconstruction on prediction results—this can be done by using several other delay times, including those to represent seasonal or annual separation of data; and (5) identification of the predictability horizon (lead time up to which reliable predictions can be made) by making predictions for different and increasing lead times. There are also other aspects that need careful consideration in advancing catchment classification and predictions in ungauged basins. For instance, regionalization based on similar catchment physical properties may not be adequate to transfer the model information due to the model and data uncertainties; rather, identification of the directionality (i.e., which catchments are better suited as donor catchments, and which are better as acceptor catchments) can be an important factor in determining the success of parameter transfer [72]. The present study, however, has made no attempt to link the prediction and classification results achieved to catchment physical properties and parameter transferability. Research in these directions is currently underway, details of which will be reported elsewhere.

## Figures and Tables

**Table 1:** Geographic distribution and physical characteristics of the 218 stations used in this study.

Jurisdictions	Major Climate Zones	Number of Stations	Average Catchment Area (km^2^)	Smallest Catchment Area (km^2^)	Largest Catchment Area (km^2^)	Average Record Length (Years)	Average Elevation (m)	Average Stream Length (m)
New South Wales (NSW)	Temperate	30	1899	14.40	35,238.90	53	825	1021
Australian Capital Territory (ACT)	Temperate	5	1406	130.00	5158.30	49	1201	983
Victoria (VIC)	Temperate	71	436	4.50	5505.80	43	703	361
Queensland (QLD)	Equatorial, Tropical, Subtropical, Grassland, Temperate	60	1662	6.60	22,885.30	44	559	1218
South Australia (SA)	Temperate, Desert	10	35,472	5.30	232,846.30	38	292	24,484
Western Australia (WA)	Temperate, Tropical	14	474	14.10	1829.40	42	289	256
Tasmania (TAS)	Temperate	12	332	18.30	775.30	49	649	256
Northern Territory (NT)	Tropical, Desert, Grassland	16	7899	95.60	47,651.50	42	285	4364

**Table 2:** Some important characteristics of the daily streamflow data used in this study.

Statistics	Minimum	Maximum	Station ID
Mean (MLD) *	0.0127	5.721	minimum: #A0030501 (SA)
			maximum: #112002A (QLD)
Standard deviation (MLD)	0.0395	13.55	minimum: #616013 (WA)
			maximum: #108003A (QLD)
Coefficient of variation	0.554	14.014	minimum: #226222 (VIC)
			maximum: #137101A (QLD)
Skewness	1.86	43.18	minimum: #229650A (VIC)
			maximum: #915011A (QLD)
Kurtosis	6.88	2795.15	minimum: #229650A (VIC)
			maximum: #915011A (QLD)
Minimum (MLD)	0	0.677	minimum: 140 stations
			maximum: #225020A (QLD)
Maximum (MLD)	0.85	541.51	minimum: #A2390523 (SA)
			maximum: #122004A (QLD)
Percentage of zeros (%)	0	81.37	minimum: 78 stations
			maximum: #G0060005 (NT)

* Million liters per day.

## Data Availability

The streamflow data used in this study were obtained from the Bureau of Meteorology, Australia. The data may be obtained from the authors upon request.

## Appendix A

### Appendix A.1. Normalized Root Mean Square Error

The root mean square error (RMSE) is one of the most commonly used error statistics for model evaluation [73,74,75]. The RMSE is defined as follows:

$$RMSE=\sqrt{\frac{\sum_{i=1}^{n}{\left(X_{i}-X_{i}^{’}\right)}^{2}}{\sum_{i=1}^{n}{\left(X_{i}-\bar{X_{i}}\right)}^{2}}}$$

$$\;\;\;\;\;\;\;\;\;\;\;\;\;\;\;\;\;\;\;\;\;\;\;\;\;\;\bar{X}=\frac{\sum_{i=1}^{n}X_{i}}{n}$$

where X_i_ is the observed value, X_i_^′^ is the predicted value, $\bar{X}$ is the mean of observed values, and n is the number of data points predicted. An RMSE value of 0 indicates a perfect match between the observed and predicted values [75], and predictions are inferior to the constant mean value if RMSE > 1. Although it is commonly accepted that the lower the RMSE, the better the model performance, Singh et al. [74] state that RMSE values less than half the standard deviation of the observed data are considered low and are appropriate for model evaluation. In the present study, a normalized version of the RMSE is used for evaluating the prediction accuracy, given by the following equation:

$$NRMSE=\frac{RMSE}{Max\left(Observedvalue\right)-Min(Observedvalue)}$$

### Appendix A.2. Correlation Coefficient

The correlation coefficient is an index of the degree of linear relationship between the observed and predicted values [75]. The accuracy of any prediction can be evaluated by using the linear correlation coefficient (CC) between the observed and predicted values, given by:

$$\;\;\;\;\;\;\;\;\;\;\;\;CC=\frac{n\left(\sum_{i=1}^{n}X_{i}X_{i}^{’}\right)-\left(\sum_{i=1}^{n}X_{i}\right)\left(\sum_{i=1}^{n}X_{i}^{’}\right)}{\sqrt{\left[n\sum_{i=1}^{n}X_{i}^{2}-\left(\sum_{i=1}^{n}X_{i}\right)\right]\left[n\sum_{i=1}^{n}{X_{i}^{’}}^{2}-{\left(\sum_{i=1}^{n}X_{i}^{’}\right)}^{2}\right]}}$$

The correlation coefficient ranges from −1 to 1. A correlation coefficient of zero indicates that there is no linear relationship between the observed and predicted values, whereas a coefficient of 1 or −1 indicates a perfect positive or negative linear relationship [75].

### Appendix A.3. Coefficient of Determination

The coefficient of determination (R^2^) describes the degree of collinearity between the observed and predicted values, and it indicates the proportion of the variance in the observed data explained by the model [75]. The coefficient of determination can be expressed as the following:

$$\;\;\;\;\;\;\;\;\;\;\;\;\;\;\;\;\;\;\;\;\;R^{2}=1-\frac{\sum_{i=1}^{n}{\left(X_{i}-X_{i}^{’}\right)}^{2}}{\sum_{i=1}^{n}{\left(X_{i}-\bar{X_{i}}\right)}^{2}}$$

The value of R^2^ ranges from 0 to 1, with higher values indicating less error variance, and values greater than 0.5 are considered acceptable [76,77]. The coefficient of determination is widely used for model evaluation; however, this coefficient is oversensitive to extreme values (outliers) and insensitive to additive and proportional differences between observed and predicted values [78].

### Appendix A.4. Nash-Sutcliffe Efficiency

The Nash-Sutcliffe efficiency (NSE) is a normalized statistic that determines the relative magnitude of the residual variance compared to the measured data variance [79]. It can be used to evaluate the predictive power of hydrologic models. The Nash-Sutcliffe efficiency is defined as follows:

$$NSE=1-\frac{\sum_{i=1}^{n}{\left(X_{i}-X_{i}^{’}\right)}^{2}}{\sum_{i=1}^{n}{\left(X_{i}-\bar{X_{i}}\right)}^{2}}$$

The NSE can range from −∞ to 1, with values between 0 and 1 generally indicating acceptable levels of performance. An NSE = 1 indicates a perfect match between the observed and predicted values, whereas an NSE = 0 means that the predicted values are as accurate as the observed values. An NSE value less than zero occurs when the mean observed value is a better predictor than the predicted value. The NSE is sensitive to extreme values and may produce sub-optimal results for datasets containing large numbers of outliers [79].
